# Effects of perfluorooctane sulfonate on genes controlling hepatic fatty acid metabolism in livers of chicken embryos

**DOI:** 10.1007/s11356-018-2358-7

**Published:** 2018-06-02

**Authors:** Annette V. Jacobsen, Marcus Nordén, Magnus Engwall, Nikolai Scherbak

**Affiliations:** 1 School of Biomedical Sciences, Charles Sturt University, Wagga Wagga, Australia; 2 The Life Science Center, School of Science and Technology, Örebro University, Örebro, Sweden; 3 Present Address: The Walter and Eliza Hall Institute, Department of Medical Biology, The University of Melbourne, Parkville, Australia; 4 MTM Research Center, School of Science and Technology, Örebro University, Örebro, Sweden; 5 Present Address: Clinical Neurochemistry Laboratory, Sahlgrenska University Hospital, Mölndal, Sweden

**Keywords:** Perfluorooctane sulfonate, PFOS, *In ovo*, Chicken, Beta oxidation, qPCR array

## Abstract

Per- and polyfluoroalkyl substances (PFAS) are synthetic surfactants with a wide variety of applications; however, due to their stability, they are particularly resistant to degradation and, as such, are classed as persistent organic pollutants. Perfluorooctane sulfonate (PFOS) is one such PFAS that is still detectable in a range of different environmental settings, despite its use now being regulated in numerous countries. Elevated levels of PFOS have been detected in various avian species, and the impact of this on avian health is of interest when determining acceptable levels of PFOS in the environment. Due to its similarities to naturally occurring fatty acids, PFOS has potential to disrupt a range of biological pathways, particularly those associated with lipid metabolism, and this has been shown in various species. In this study, we have investigated how in ovo exposure to environmentally relevant levels of PFOS affects expression of genes involved in lipid metabolism of developing chicken embryos. We have found a broad suppression of transcription of genes involved in fatty acid oxidation and PPAR-mediated transcription with more significant effects apparent at lower doses of PFOS. These results highlight the need for more research investigating the biological impacts of low levels of PFAS to properly inform environmental policy governing their regulation.

**Electronic supplementary material:**

The online version of this article (10.1007/s11356-018-2358-7) contains supplementary material, which is available to authorized users.

## Introduction

The presence of persistent organic pollutants (POPs) is ever increasing in the environment due to technological development and growing commercialization of synthetic compounds. Per- and polyfluoroalkyl substances (PFAS) are anthropogenic compounds used commercially for their stability and surfactant properties. One of the major PFAS is perfluorooctane sulfonate (PFOS), which consists of a perfluorinated eight-carbon backbone and a sulphonate group. This makes this PFAS amphiphilic and a good surfactant with many fields of application. PFAS are ideal flame-retardants because of their carbon-fluorine bond, which is one of the strongest in organic chemistry. This is also what renders them resilient to biological degradation, strong acids and alkalis and photolysis. Industrial and commercial uses include stain repellent, non-stick coatings and flame retardants for example in firefighting foam, clothes and upholstery, cooking utensils, food wrappers and electronics (Renner 2001).

The chemical and biological stability explains measurable environmental levels of different PFAS (i.e. PFOS) in different matrices all over the globe. PFOS can be found in humans worldwide (Kärrman et al. 2007), and is detected far from manufacturing facilities in environments such as in high arctic icecaps, high-altitude lakes, off-shore waters, and deep-sea oceans. PFOS is detectable in various animal species (Houde et al. 2011), and is found in high concentrations in top predator avian species. A study of eggs and developing embryos of the great cormorant and herring gull showed PFOS concentrations in the μg/g wet weight in whole egg and liver (Nordén et al. 2013). Studies of the effects of environmentally comparable concentrations of PFOS on development of White Leghorn chicken showed reduced embryo survival, significant immunological, neurological and morphological changes in treated embryos compared to controls (Nordén et al. 2016; Peden-Adams et al. 2009). PFOS-induced immunotoxicity has been noted in various species at environmentally relevant doses (DeWitt et al. 2012), and has the potential to impact the fitness of wild species, particularly if faced with environmental challenges such as infection.

Hepatotoxicity is another important feature of PFOS exposure, with hepatomegaly, necrosis, and lipid accumulation found in various animal models (Bijland et al. 2011; Du et al. 2009; Peden-Adams et al. 2009; Wan et al. 2012; Wang et al. 2014), although the mechanism behind this is still not clear. Transcriptomic analysis of the livers of 6-week-old chicken (*Gallus gallus*) that were subcuticularly exposed to low doses of PFOS showed changes in expression of genes mainly involved in electron and oxygen transport, and the metabolism of lipids and fatty acids (Yeung et al. 2007). Other studies have also assessed changes to gene expression using microarrays (Martin et al. 2007; O'Brien et al. 2011), or investigation of small subsets of genes (Cwinn et al. 2008; O'Brien et al. 2009); however, these have given conflicting results regarding how PFOS impacts lipid metabolism. Moreover, direct comparison of these studies is challenging, due to large variations in the dose of PFOS used, with many studies using levels well in excess of that found environmentally. Despite the varied results in the literature, hepatic steatosis is one of the more commonly documented effects of PFOS exposure, which strongly suggests liver lipid metabolism is indeed disrupted. To address how gene expression changes contribute to this, approaches with greater sensitivity than conventional microarrays, and greater depth and resolution than smaller focused gene studies, are required. In the current study, we investigated the effects of environmentally relevant concentrations of PFOS on expression of genes controlling liver fatty acid metabolism of chicken embryos using the Chicken Fatty Acid Metabolism RT2 profiler PCR array®.

## Material and methods

### Egg incubation and exposure

Treatments were done as previously described by Nordén et al. (2016). Fertilised, unincubated eggs from White Leghorn chicken (*Gallus gallus*) were purchased from Ova Production, Vittinge, Sweden and kept at 10–12 °C until incubation. Potassium salt of PFOS (Chemica 98%, Lot 77.282, approximately 21% branched isomer) was dissolved in 5% solution of dimethyl sulfoxide (DMSO; Sigma-Aldrich, Darmstadt, Germany) to final concentrations of either 0.1 or 1.0 mg/ml. On the fourth day of incubation, a single microinjection of 1 µl of this PFOS solution per gram of egg was aseptically added into air sacs of eggs resulting in treatment concentrations of 0.1 respectively 1.0 µg of PFOS per 1g of egg. All treatments were done in four replicates. Eggs injected with 1 µl/g egg of DMSO only (5%; *n* = 4) were used as controls. Holes were sealed with paraffin and eggs put in an incubator at 37.5 °C and 60% humidity. Eggs were turned in a 6-h cycle and sacrificed 1 day before expected pipping, i.e. 19 days post incubation start. Obtained liver samples were preserved at − 80 °C in RNA stabilisation solution (RNAlater®, Invitrogen/ThermoFisher, MA, USA) until use. The experimental protocol was approved by the Swedish Board of Agriculture, Jönköping, Sweden.

### RNA purification and qPCR

Approximately 15 mg of chicken embryo liver tissue was used for RNA purification using RNeasy® mini kit (QIAGEN, Hilden, Germany) according to manufacturer protocol. RNA was checked for purity and quantified using a spectrophotometer (NanoDrop® 2000; NanoDrop Technologies, Wilmington, DE). RNA integrity was confirmed with gel electrophoresis using a 1.2% (*w*/*v*) agarose gel. Complementary DNA (cDNA) was synthesised using RT2 First Strand Kit® (QIAGEN) according to manufacturer instructions using 0.5 μg purified RNA from each sample. Using qPCR, samples were analysed using Chicken Fatty Acid Metabolism RT2 profiler PCR array® (Catalogue PAGG-007Z; QIAGEN). These arrays come in 96-well plate format, which include 84 wells containing primers for genes of interest, 5 wells containing housekeeping genes suggested by QIAGEN, and additional controls to analyse genomic DNA contamination, reverse transcription efficiency and PCR array reproducibility. For the list of analysed genes, see Table S1 in Supporting information, and for information on the RT2 profiler system see https://dataanalysis.sabiosciences.com/pcr/documents/RT2ProfilerDataAnalysisHandbook.pdf.

One plate was used to analyse each biological sample, and an electronic pipetting system was used to limit any variation caused by pipetting technique. The qPCR program was set to 40 cycles consisting of the following temperatures and time intervals: an initial denaturation at 95 °C for 5 minutes, followed by 15 s at 95 and 60 °C for 1 min for 40 cycles using an Applied Biosystems® 9700 thermocycler (Applied Biosystems, Carlsbad, CA). Each run was completed with melting curve analysis to confirm a single amplified product.

### Data analysis

The analysis software RT2 Profiler PCR array Data Analysis® (QIAGEN) version 3.5 was used for interpretation of PCR array data. In brief, data was first normalised against the geometric mean of a panel of housekeeping genes suggested by QIAGEN to generate a ΔCt value. As the differences between the geometric means of all control and test groups were within the limits suggested by QIAGEN, we opted to use all five genes in the panel for normalisation (*ACTB*, *H6PD*, *HMBS*, *RPL4* and *UBC*). The software then calculates an average ΔCt value for each of the control and treatments groups, as well as a standard deviation (SD) to assess variability. The average ΔCt values are used to calculate fold change (FC), which is the ratio of relative gene expression between the control group and the test group, using the formula 2^(ΔΔCt), where ΔΔCt = average ΔCt(test group) – average ΔCt(control group). For the purposes of this study, FC is represented as fold regulation (FR), where for FC ≥ 1, FR = FC and for FC < 1, FR = − 1/FC. The *p* values for each gene were determined using a Student’s *t* test, which was calculated using the average ΔCt of each test group versus the control group and their associated SD. To focus on gene expression changes that were more likely to be associated with a biological effect, we used a cut-off for differential expression as a FR ± 2 (*p* ≤ 0.05).

KEGG pathway analysis of all tested genes, and those differentially expressed genes at either dose of PFOS, was performed using STRING version 10.5. During the pathway analysis, STRING performs a Fisher’s exact test based on the number of specified genes that fall within a particular pathway category, the number of total genes annotated to that pathway and the total gene number present in the organism being studied. This is then corrected for multiple testing to give a false discovery rate, which is a measure of the likely proportion of false positive gene matches for the specified pathway (Szklarczyk et al. 2017).

## Results

**Fig. 1:**
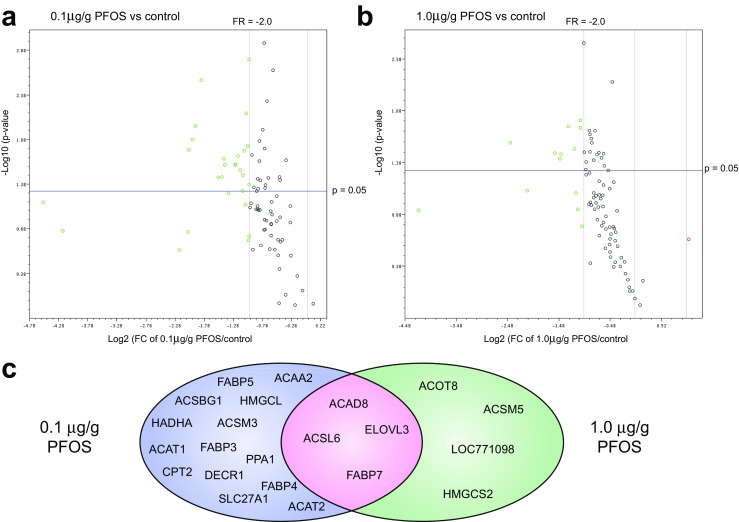
Results of gene expression analysis from the Chicken Fatty Acid Metabolism RT2 profiler PCR array®. Panel **a**, **b** shows volcano plots depicting a general downregulation of studied genes after in ovo treatment with 0.1 μg/g (**a**) and 1.0 μg/g (**b**) of PFOS. Genes with greater than two-fold regulation (FR) in expression are shown in green (suppression) and red (induction). Genes with significant (*p* ≤ 0.5) changes to expression are positioned above the horizontal line. Panel **c** shows comparison genes with altered expression (FR ≥ ± 2; *p* ≤ 0.5) by treatment with 0.1 μg/g (purple) and 1.0 μg/g (green) of PFOS. Genes whose expression was altered at both doses are represented by the intersection (pink)

As previous studies indicated dysregulated lipid metabolism after treatment with perfluoronated compounds, we used a focused PCR array to analyse 84 genes associated with lipid metabolism (Table S1). Normalisation against the five reference genes (*ACTB*, *H6PD*, *HMBS*, *RPL4*, *UBC*) revealed a general downregulation of expression after treatment of eggs with PFOS at both 0.1 and 1.0 μg/g of egg (Fig. 1a, b). Of these 84 genes, we found 22 genes with significant downregulation (fold regulation (FR) ≤ − 2; *p* ≤ 0.05), with four of these genes (*ACAD8*, *ACSL6*, *ELOVL3*, *FABP7*) downregulated in both the 0.1 and 1.0 μg/g treatment groups (Fig. 1c; Table 1). A further seven genes with FR ≤ − 2 (*p* ≤ 0.05) at a PFOS dose 0.1 μg/g of egg (*ACAA2*, *ACAT1*, *ACSM3*, *CPT2*, *DECR1*, *FABP3*, FABP5) were also downregulated to a lesser extent (FR ≤ − 1.5; *p* ≤ 0.05) at the higher dose of PFOS (Table 1; FR ≤ − 1.5 and > − 2.0 shown in italics). A similar effect was seen with *ACOT8* and *LOC771098*, although they were both more strongly affected by the 1.0 μg/g of egg dose of PFOS (Table 1). Additionally, ACAT2 (FR = − 2.68; *p* = 0.025; 0.1 μg/g PFOS) was also downregulated by 1.0 μg/g PFOS (FR = − 2.22; *p* = 0.08).

**Table 1:** Genes with expression changes of ≥ 2 (*p* ≤ 0.05) after treatment with PFOS. Expression changes of significantly regulated genes in both treatment conditions. Values in italics indicate significant (*p* ≤ 0.5) expression changes, with fold regulation < 2. Values in grey indicate where there were no statistically significant (*p* > 0.5) changes to expression

Gene symbol	PFOS 0.1 μg/g egg		PFOS 1.0 μg/g egg	
	fold regulation	*P* value	Fold regulation	*P* value
ACAA2	− 2.0106	0.00168	*− 1.9936*	*0.002946*
ACAD8	− 2.3604	0.02053	− 2.2698	0.030889
ACAT1	− 2.3008	0.020093	*− 1.9327*	*0.041243*
ACAT2	− 2.6831	0.025219	− 2.2171	0.082354
ACOT8	*− 1.7545*	*0.03791*	− 2.084	0.01941
ACSBG1	− 2.37	0.025374	− 1.39^A^	0.340918^A^
ACSL6	− 3.954	0.013228	− 2.9527	0.03381
ACSM3	− 2.1211	0.017689	*− 1.7454*	*0.039628*
ACSM5	− 1.4078	0.891541	− 5.3785	0.020546
CPT2	− 2.0424	0.015545	*− 1.8386*	*0.032922*
DECR1	− 2.0763	0.006687	*− 1.8092*	*0.024563*
ELOVL3	− 3.8073	0.009259	− 2.715	0.034701
FABP3	− 2.236	0.029092	*− 1.9463*	*0.048893*
FABP4	− 4.1192	0.017246	− 1.73^a^	0.118269^A^
FABP5	− 3.5536	0.002826	*− 1.8447*	*0.022399*
FABP7	− 2.904	0.035071	− 2.7541	0.038439
HADHA	− 2.0065	0.042016	− 1.6934	0.087043
HMGCL	− 2.7223	0.021643	− 1.6228	0.06323
HMGCS2	− 1.1307	0.441867	− 2.455	0.033322
LOC771098	*− 1.5118*	*0.002207*	− 2.0853	0.016339
PPA1	− 2.774	0.034539	− 1.5339	0.158511
SLC27A1	− 2.1635	0.033117	− 1.6206	0.085314

^a^Analysed data had C_t_ > 30 so results should be interpreted with caution

The genes selected for our analysis were those specifically targeted to metabolic pathways, so we performed enrichment analyses to determine which pathways were most influenced by our selected genes (Table S2). Of these pathways, a subset showed a proportionally large number of genes affected by treatment with either dose of PFOS, particularly butanoate metabolism, PPAR signalling pathway, fatty acid degradation, valine, leucine and isoleucine degradation and fatty acid metabolism (Table 2). Full expression data for all genes tested in the PCR array can be found in Table S3.

**Table 2:** Signalling pathways affected by PFOS treatment. KEGG pathway information generated from analysis in STRING using genes whose expression showed ≥ two-fold regulation (*p* ≤ 0.05) at either of the two administered doses of PFOS, including the proportion of genes analysed within these pathways that met the aforementioned criteria. Fraction of affected genes refers to the number of differentially affected genes compared to the total number of genes in that pathway that were analysed in the array (refer Table S2). The false discovery rate (calculated by STRING) is an indication of the likely proportion of false positive gene matches for the specified pathway

KEGG ID	Pathway description	Observed genes	Fraction of affected genes	False discovery rate
650	Butanoate metabolism	ACAT1, ACAT2, ACSM3, ACSM5, HMGCS2, HADHA, HMGCL	7 of 13	6.43E-14
3320	PPAR signalling pathway	ACSBG1, ACSL6, CPT2, FABP3, FABP4, FABP5, FABP7, SLC27A1	8 of 28	2.01E-13
71	Fatty acid degradation	ACAA2, ACAT1, ACAT2, ACSBG1, ACSL6, CPT2, HADHA	7 of 22	3.28E-13
280	Valine, leucine and isoleucine degradation	ACAA2, ACAD8, ACAT1, ACAT2, HMGCS2, HADHA, HMGCL	7 of 14	1.73E-12
1212	Fatty acid metabolism	ACAA2, ACAT1, ACAT2, ACSBG1, ACSL6, CPT2, HADHA	7 of 27	2.00E-12
72	Synthesis and degradation of ketone bodies	ACAT1, ACAT2, HMGCS2, HMGCL	4 of 7	7.63E-09
1100	Metabolic pathways	ACAA2, ACAD8, ACAT1, ACAT2, ACOT8, ACSBG1, ACSL6, ACSM3, ACSM5, HMGCS2, HADHA, HMGCL	12 of 40	9.17E-09
900	Terpenoid backbone biosynthesis	ACAT1, ACAT2, HMGCS2	3 of 4	2.22E-05
640	Propanoate metabolism	ACAT1, ACAT2, HADHA	3 of 8	0.000115
380	Tryptophan metabolism	ACAT1, ACAT2, HADHA	3 of 5	0.000188
1120	Microbial metabolism in diverse environments	ACAA2, ACAT1, ACAT2, HADHA	4 of 10	0.000188
310	Lysine degradation	ACAT1, ACAT2, HADHA	3 of 5	0.000237
4146	Peroxisome	ACOT8, ACSL6, HMGCL	3 of 16	0.00107
1200	Carbon metabolism	ACAT1, ACAT2, HADHA	3 of 8	0.0017
62	Fatty acid elongation	ACAA2, HADHA	2 of 4	0.00271
630	Glyoxylate and dicarboxylate metabolism	ACAT1, ACAT2	2 of 4	0.00456
620	Pyruvate metabolism	ACAT1, ACAT2	2 of 6	0.00917
4920	Adipocytokine signalling pathway	ACSBG1, ACSL6	2 of 13	0.0213

## Discussion

PFAS such as PFOS are persistent environmental pollutants and well known to cause adverse effects on the health of various wild and laboratory animals. Although most European and Northern American countries now regulate production of these compounds, they are still actively used in other countries, such as China (Fu et al. 2016), and are found to be present in a range of consumer products (Kotthoff et al. 2015). Acceptable environmental levels have been debated, with a recent push to adopt lower thresholds from a number of different agencies. Additionally, recent animal experiments indicate PFAS doses corresponding to current environmental levels can impact various biological pathways (Lilienthal et al. 2017). In this study, we have used qPCR arrays to examine the effect of PFOS on expression of genes related to lipid metabolism in livers of chicken embryos, and have found that low doses suppress transcription of genes relating to lipid catabolism and fatty acid β-oxidation.

By using KEGG pathway analysis, the top identified metabolic process affected in our analysis was butanoate metabolism, which involves processing of short chain fatty acids (SCFAs) and is known to be important for regulating mitochondrial energy production, lipogenesis and cellular metabolic processes including fatty acid oxidation (Schönfeld and Wojtczak 2016). Additionally, SCFAs, including butyrate, have been shown to act as a switch between fatty acid oxidation and lipogenesis in a PPARγ-dependent manner (den Besten et al. 2015). Deregulated lipogenesis in the form of hepatic steatosis is commonly seen after exposure to PFOS (Cheng et al. 2016; Lai et al. 2017), as are perturbations to fatty acid oxidation (Wan et al. 2012). However, laboratory results are contradictory in relation to this, with some studies indicating increased beta oxidation or gene expression of relevant enzymes (Hu et al. 2005; Nordén et al. 2012; Tan et al. 2012), while others indicate beta oxidation is supressed (Adinehzadeh and Reo 1998; Bijland et al. 2011; Cheng et al. 2016). These differences may be a result of the range of concentrations being used, different responses between animal models and cell culture and differences in how the pathways themselves are assessed. Interestingly, our results indicate a suppression of transcription of genes involved in beta oxidation that is more apparent at lower doses corresponding to environmentally relevant concentrations, suggesting that metabolic responses to PFOS could differ based on the level of exposure.

We also noted a number of the genes found to be differentially expressed in this study relate to mitochondrial beta oxidation. This includes *CPT2* and *DECR1*, which both have crucial roles in positive regulation of this pathway, as well as other positive regulators of beta oxidation, such as *HADHA*, *ACAA2* and *ACAT1* (Houten et al. 2016). These were all found to be downregulated at the lower dose of 0.1 μg/g egg of PFOS (Table 1). Previously, induction of beta oxidation by PFOS has previously been linked to peroxisomal beta oxidation (Hu et al. 2005; Tan et al. 2012); however, Wan et al. (2012) showed that mice exposed to PFOS had both increased peroxisomal beta oxidation and decreased mitochondrial beta oxidation. Importantly, impaired mitochondrial function is proposed as a key event leading to hepatic steatosis (Angrish et al. 2016), such as is seen in PFOS liver toxicity. Moreover, Wan et al. (2012) also found an increase in total beta oxidation, similar to that found in our previous analysis of day 10 embryonic chicken livers (Nordén et al. 2012). Interestingly, the only acyl-CoA thioesterase (ACOT) found to be downregulated by PFOS (at 1.0 μg/g of egg) in this current study was *ACOT8*, which is proposed to be the predominant ACOT involved in negative regulation of peroxisomal beta oxidation (Hunt et al. 2014). Together, these data imply PFOS may induce a transition from mitochondrial beta oxidation to peroxisomal beta oxidation, which could help to clarify both the mechanism of PFOS toxicity and explain some of the contradictory results found in the literature, including our own previous results. Analysis of the gene expression profile of day 10 embryonic livers would need to be done to clarify if this was the case, or whether the differences between the two studies were due to different transcriptional responses induced by PFOS at those developmental time points.

The broad transcriptional repression seen in this study could be explained by PFOS binding to, and interfering with, relevant transcription factors. One possible mechanism suggested by the KEGG pathway analysis in our study is PPAR-mediated regulation, and indeed there are a number of studies that implicate PPAR as being responsible for the metabolic disruption seen after PFOS exposure (Cwinn et al. 2008; Fang et al. 2012; Lai et al. 2017), although, like beta-oxidation, the directionality of this response is still debated. Interestingly, the second most significantly affected pathway in our KEGG analysis was PPAR signalling, with approximately one third of the genes tested being significantly repressed by two-fold or more. Although there are fewer studies investigating the links between PPAR and beta oxidation in chicken, there is indication that there are some similarities to other model organisms. In particular, carnitine palmitoyltransferase 1a (*CPT1A*) transcription seems to be regulated through PPARα (Honda et al. 2016). Similar studies of PPAR-related effects of PFOS on the embryonic chicken liver have given varied responses, with studies in both 18 and 21 days chicken embryos indicating no significant changes to expression of PPAR-induced genes (O'Brien et al. 2009; Strömqvist et al. 2012). It should be noted, however, that the study conducted at the same time point as ours used doses of PFOS 20-fold higher than the upper concentration used in the present study.

In rodent and chicken studies, and in cell culture models of various species, most studies have found that PFOS causes induction of PPAR-mediated transcription, particularly *PPARα* (Bjork et al. 2011; Elcombe et al. 2012; Strömqvist et al. 2012). Interestingly, a study by Wang et al. (2014) showed that both *PPARα* and *CPT1A* gene expression were impaired by PFOS only in mice that were fed a high-fat diet. This observation may have relevance to studies such as ours, considering the relatively high in ovo fat content, and may also go partway toward explaining differing results seen both between and within different species models. Furthermore, both avian and rodent studies indicate that PPAR-mediated effects are not solely responsible for toxic and disruptive effects of PFOS (Abbott et al. 2009; O'Brien et al. 2009; Rosen et al. 2010), indicating other transcription factors may be involved. One particularly interesting candidate is hepatocyte nuclear factor 4α (*HNF*-*4α*), a transcription factor with known effects on lipid metabolism through regulating both *PPARα* and *CPT1A* gene transcription (Karagianni and Talianidis 2015; Martinez Jimenez et al. 2010). Binding of PFOS to *HNF*-*4α* is believed to disrupt the normal lipid binding required for its stabilisation, with PFOS treatment inducing degradation of both the mouse and human proteins (Beggs et al. 2016). The ability of PFOS to induce degradation of *HNF*-*4α*, or other transcription factors, could help explain the downregulation of multiple genes related to lipid metabolism seen here. Moreover, knockout of *HNF*-*4α* induces liver steatosis in mouse models (Hayhurst et al. 2001), similar to that seen after PFOS treatment. Follow-up studies would be required to see whether this effect was also seen in avian species.

Our results indicate broad suppression of transcription of genes associated with lipid metabolism after in ovo exposure of chicken embryos to PFOS, particularly at lower, environmentally relevant doses. There are, however, some limitations of these results. Firstly, we only analysed two environmentally relevant doses of PFOS. This decision was made based on our previous work, which suggested that these doses were sufficient to cause changes to lipid metabolism (Nordén et al. 2012). However, in the previous study, we noted the most profound effect at 0.3 μg/g of egg dose of PFOS, an intermediate dose to those used here. As most of the statistically significant changes to gene expression noted in this study were relatively small, we may have found more definitive results if that dose had also been used in this study. Secondly, due to the cost of the arrays, we were only able to analyse four individuals per treatment group and we were not able to perform duplicate plates. However, this is a robust commercially designed assay that has been used in a wide range of studies, and is equipped with various controls, including PCR reproducibility. This control showed little variability both within and between plates, giving us confidence in the assay and the results it generated. Moreover, the fact that the responses were consistent enough to give statistically significant data despite the small sample size is encouraging. Thirdly, as we were unable to determine the sex of the embryos studied, we cannot exclude that sex differences have contributed to these results. Follow up studies will need to take this into account. Lastly, it should also be noted that we only analysed gene expression data and, as such, further studies, such as metabolomics or proteomics, are required to confirm whether these changes relate to a functional impairment of lipid metabolism.

For these results to have relevance in relation to wild bird species found to be affected by PFOS, we would also suggest investigating whether similar effects are seen in other avian species. As we do not yet have full coverage of the genomes of wild avian species, a metabolomics-based study would presently be the most appropriate method to investigate this. That said, the Avian Phylogenomics Consortium is currently working to sequence all known avian species (Zhang 2015), and this knowledge would enable comparative gene expression studies between species. If similar results as seen here are found in wild avian species, changes to expression of key metabolic enzymes such as CPT2 and DECR1 could potentially act as environmental markers of PFOS exposure. Such applications would, however, need to take into account whether other common pollutants have overlapping effects.

A better understanding of species-specific effects of PFAS and the doses at which they occur is important when considering both acceptable levels of these compounds in the environment and safe exposure levels for persons with occupational contact with PFAS. Although we did not directly measure hepatic liver PFOS concentrations, similarly designed previous studies have shown these concentrations are approximately equivalent to the administered dose at the time point we measured (Nordén et al. 2016; O'Brien et al. 2009). As these concentrations are comparable to those found in environmental analyses of wild birds, it is important that future studies are done to determine whether similar effects are seen in these species, and whether chicken can continue to be used as a model for environmental exposure. Particularly, since our current study indicates more profound effects on expression of genes related to lipid metabolism at lower doses, we would suggest that current environmental levels are considered when planning any studies investigating physiological effects of PFAS.

## Conclusion

In this study, we investigated the influence of perfluorooctane sulfonate (PFOS) on genes associated to fatty acid metabolism in developing chicken embryos. Liver samples from embryos treated with PFOS showed downregulation of the majority of genes involved in metabolism of fatty acids and this effect was more pronounced at the lower of the two tested doses of PFOS. Our findings shows that environmentally relevant concentrations of perfluorooctane sulfonate could impact energy metabolism in livers of developing chicken embryos, and suggest further functional studies should be performed to confirm the physiological impact of this.

## Electronic supplementary material

Table S1 List of genes included in RT2 Profiler QPCR array fatty acid metabolism 96-well plate. (XLSX 11 kb)

Table S2 List of KEGG pathways targeted by our analysis; generated by running the list of all genes in Table S1 through a STRING pathway analysis. (XLSX 28 kb)

Table S3 Complete expression data generated from the RT2 Profiler QPCR array after normalisation against supplied control genes. (XLSX 17 kb)

## References

[CR1] Abbott BD, Wolf CJ, Das KP, Zehr RD, Schmid JE, Lindstrom AB, Strynar MJ, Lau C (2009). Developmental toxicity of perfluorooctane sulfonate (PFOS) is not dependent on expression of peroxisome proliferator activated receptor-alpha (PPAR alpha) in the mouse. Reprod Toxicol.

[CR2] Adinehzadeh M, Reo NV (1998). Effects of peroxisome proliferators on rat liver phospholipids: sphingomyelin degradation may be involved in hepatotoxic mechanism of perfluorodecanoic acid. Chem Res Toxicol.

[CR3] Angrish MM, Kaiser JP, McQueen CA, Chorley BN (2016). Tipping the balance: hepatotoxicity and the 4 apical key events of hepatic steatosis. Toxicol Sci.

[CR4] Beggs K, McGreal S, McCarthy A, Gunewardena S, Lampe J, Lau C, Apte U (2016). The role of hepatocyte nuclear factor 4-alpha in perfluorooctanoic acid- and perfluorooctanesulfonic acid-induced hepatocellular dysfunction. Toxicol Appl Pharmacol.

[CR5] Bijland S, Rensen PCN, Pieterman EJ, Maas ACE, van der Hoorn JW, van Erk MJ, Havekes LM, Willems van Dijk K, Chang SC, Ehresman DJ, Butenhoff JL, Princen HMG (2011). Perfluoroalkyl sulfonates cause alkyl chain length–dependent hepatic steatosis and hypolipidemia mainly by impairing lipoprotein production in APOE*3-Leiden CETP mice. Toxicol Sci.

[CR6] Bjork JA, Butenhoff JL, Wallace KB (2011). Multiplicity of nuclear receptor activation by PFOA and PFOS in primary human and rodent hepatocytes. Toxicology.

[CR7] Cheng J, Lv S, Nie S, Liu J, Tong S, Kang N, Xiao Y, Dong Q, Huang C, Yang D (2016). Chronic perfluorooctane sulfonate (PFOS) exposure induces hepatic steatosis in zebrafish. Aquat Toxicol.

[CR8] Cwinn MA, Jones SP, Kennedy SW (2008). Exposure to perfluorooctane sulfonate or fenofibrate causes PPAR-α dependent transcriptional responses in chicken embryo hepatocytes. Comparative Biochemistry and Physiology Part C: Toxicology & Pharmacology.

[CR9] DeWitt JC, Peden-Adams MM, Keller JM, Germolec DR (2012). Immunotoxicity of perfluorinated compounds: recent developments. Toxicol Pathol.

[CR10] Du Y, Shi X, Liu C, Yu K, Zhou B (2009). Chronic effects of water-borne PFOS exposure on growth, survival and hepatotoxicity in zebrafish: a partial life-cycle test. Chemosphere.

[CR11] Elcombe CR, Elcombe BM, Foster JR, Chang S-C, Ehresman DJ, Butenhoff JL (2012). Hepatocellular hypertrophy and cell proliferation in Sprague–Dawley rats from dietary exposure to potassium perfluorooctanesulfonate results from increased expression of xenosensor nuclear receptors PPARα and CAR/PXR. Toxicology.

[CR12] Fang C, Wu X, Huang Q, Liao Y, Liu L, Qiu L, Shen H, Dong S (2012). PFOS elicits transcriptional responses of the ER, AHR and PPAR pathways in Oryzias melastigma in a stage-specific manner. Aquat Toxicol.

[CR13] Fu J, Gao Y, Cui L, Wang T, Liang Y, Qu G, Yuan B, Wang Y, Zhang A, Jiang G (2016). Occurrence, temporal trends, and half-lives of perfluoroalkyl acids (PFAAs) in occupational workers in China. Sci Rep.

[CR14] Hayhurst GP, Lee YH, Lambert G, Ward JM, Gonzalez FJ (2001). Hepatocyte nuclear factor 4alpha (nuclear receptor 2A1) is essential for maintenance of hepatic gene expression and lipid homeostasis. Mol Cell Biol.

[CR15] Honda K, Saneyasu T, Sugimoto H, Kurachi K, Takagi S, Kamisoyama H (2016). Role of peroxisome proliferator-activated receptor alpha in the expression of hepatic fatty acid oxidation-related genes in chickens. Anim Sci J.

[CR16] Houde M, De Silva AO, Muir DC, Letcher RJ (2011). Monitoring of perfluorinated compounds in aquatic biota: an updated review. Environmental Science & Technology.

[CR17] Houten SM, Violante S, Ventura FV, Wanders RJA (2016). The biochemistry and physiology of mitochondrial fatty acid β-oxidation and its genetic disorders. Annu Rev Physiol.

[CR18] Hu W, Jones PD, Celius T, Giesy JP (2005). Identification of genes responsive to PFOS using gene expression profiling. Environ Toxicol Pharmacol.

[CR19] Hunt MC, Tillander V, Alexson SEH (2014). Regulation of peroxisomal lipid metabolism: the role of acyl-CoA and coenzyme a metabolizing enzymes. Biochimie.

[CR20] Karagianni P, Talianidis I (2015). Transcription factor networks regulating hepatic fatty acid metabolism. Biochimica et Biophysica Acta (BBA)—Molecular and Cell Biology of Lipids.

[CR21] Kärrman A, Langlois I, van Bavel B, Lindström G, Oehme M (2007). Identification and pattern of perfluorooctane sulfonate (PFOS) isomers in human serum and plasma. Environ Int.

[CR22] Kotthoff M, Müller J, Jürling H, Schlummer M, Fiedler D (2015). Perfluoroalkyl and polyfluoroalkyl substances in consumer products. Environ Sci Pollut Res.

[CR23] Lai KP, Li JW, Cheung A, Li R, Billah MB, Chan TF, Wong CK (2017) Transcriptome sequencing reveals prenatal PFOS exposure on liver disorders Environmental Pollution doi:10.1016/j.envpol.2017.01.041 10.1016/j.envpol.2017.01.041 28131474

[CR24] Lilienthal H, Dieter HH, Hölzer J, Wilhelm M (2017). Recent experimental results of effects of perfluoroalkyl substances in laboratory animals—relation to current regulations and guidance values. Int J Hyg Environ Health.

[CR25] Martin MT, Brennan RJ, Hu W, Ayanoglu E, Lau C, Ren H, Wood CR, Corton JC, Kavlock RJ, Dix DJ (2007). Toxicogenomic study of triazole fungicides and perfluoroalkyl acids in rat livers predicts toxicity and categorizes chemicals based on mechanisms of toxicity. Toxicol Sci.

[CR26] Martinez Jimenez C, Kyrmizi I, Cardot P, Gonzalez F, Talianidis I (2010). Hepatocyte nuclear factor 4alpha coordinates a transcription factor network regulating hepatic fatty acid metabolism. Mol Cell Biol.

[CR27] den Besten G, Bleeker A, Gerding A, van Eunen K, Havinga R, van Dijk TH, Oosterveer MH, Jonker JW, Groen AK, Reijngoud DJ, Bakker BM (2015). Short-chain fatty acids protect against high-fat diet–induced obesity via a PPARγ-dependent switch from lipogenesis to fat oxidation. Diabetes.

[CR28] Nordén M, Westman O, Venizelos N, Engwall M (2012). Perfluorooctane sulfonate increases β-oxidation of palmitic acid in chicken liver. Environ Sci Pollut Res.

[CR29] Nordén M, Berger U, Engwall M (2013). High levels of perfluoroalkyl acids in eggs and embryo livers of great cormorant (Phalacrocorax carbo sinensis) and herring gull (Larus argentatus) from Lake Vänern, Sweden. Environ Sci Pollut Res.

[CR30] Nordén M, Berger U, Engwall M (2016). Developmental toxicity of PFOS and PFOA in great cormorant (Phalacrocorax carbo sinensis), herring gull (Larus argentatus) and chicken (Gallus gallus domesticus). Environ Sci Pollut Res.

[CR31] O'Brien JM, Carew AC, Chu S, Letcher RJ, Kennedy SW (2009). Perfluorooctane sulfonate (PFOS) toxicity in domestic chicken (Gallus gallus domesticus) embryos in the absence of effects on peroxisome proliferator activated receptor alpha (PPARalpha)-regulated genes. Comparative Biochemistry and Physiology Part C: Toxicology & Pharmacology.

[CR32] O'Brien JM, Austin AJ, Williams A, Yauk CL, Crump D, Kennedy SW (2011). Technical-grade perfluorooctane sulfonate alters the expression of more transcripts in cultured chicken embryonic hepatocytes than linear perfluorooctane sulfonate. Environ Toxicol Chem.

[CR33] Peden-Adams MM, Stuckey JE, Gaworecki KM, Berger-Ritchie J, Bryant K, Jodice PG, Scott TR, Ferrario JB, Guan B, Vigo C, Boone JS, McGuinn WD, DeWitt JC, Keil DE (2009). Developmental toxicity in white leghorn chickens following in ovo exposure to perfluorooctane sulfonate (PFOS). Reprod Toxicol.

[CR34] Renner R (2001). Growing concern over perfluorinated chemicals. Environmental Science & Technology.

[CR35] Rosen MB, Schmid JR, Corton JC, Zehr RD, Das KP, Abbott BD, Lau C (2010). Gene expression profiling in wild-type and PPAR⍺-null mice exposed to perfluorooctane sulfonate reveals PPAR⍺-independent effects. PPAR Res.

[CR36] Schönfeld P, Wojtczak L (2016). Short- and medium-chain fatty acids in energy metabolism: the cellular perspective. J Lipid Res.

[CR37] Strömqvist M, Olsson JA, Kärrman A, Brunström B (2012). Transcription of genes involved in fat metabolism in chicken embryos exposed to the peroxisome proliferator-activated receptor alpha (PPARalpha) agonist GW7647 or to perfluorooctane sulfonate (PFOS) or perfluorooctanoic acid (PFOA). Comparative Biochemistry and Physiology Part C: Toxicology & Pharmacology.

[CR38] Szklarczyk D, Morris JH, Cook H, Kuhn M, Wyder S, Simonovic M, Santos A, Doncheva NT, Roth A, Bork P, Jensen LJ, von Mering C (2017). The STRING database in 2017: quality-controlled protein–protein association networks, made broadly accessible. Nucleic Acids Res.

[CR39] Tan F, Jin Y, Liu W, Quan X, Chen J, Liang Z (2012). Global liver proteome analysis using iTRAQ labeling quantitative proteomic technology to reveal biomarkers in mice exposed to perfluorooctane sulfonate (PFOS). Environmental Science & Technology.

[CR40] Wan HT, Zhao YG, Wei X, Hui KY, Giesy JP, Wong CK (2012). PFOS-induced hepatic steatosis, the mechanistic actions on beta-oxidation and lipid transport. Biochim Biophys Acta Gen Subj.

[CR41] Wang L, Wang Y, Liang Y, Li J, Liu Y, Zhang J, Zhang A, Fu J, Jiang G (2014). PFOS induced lipid metabolism disturbances in BALB/c mice through inhibition of low density lipoproteins excretion. Sci Rep.

[CR42] Yeung LW, Guruge KS, Yamanaka N, Miyazaki S, Lam PK (2007). Differential expression of chicken hepatic genes responsive to PFOA and PFOS. Toxicology.

[CR43] Zhang G (2015). Bird sequencing project takes off. Nature.

